# Accelerating the annotation of sparse named entities by dynamic sentence selection

**DOI:** 10.1186/1471-2105-9-S11-S8

**Published:** 2008-11-19

**Authors:** Yoshimasa Tsuruoka, Jun'ichi Tsujii, Sophia Ananiadou

**Affiliations:** 1 School of Computer Science, The University of Manchester, MIB, 131 Princess Street, Manchester, M1 7DN, UK; 2 National Centre for Text Mining (NaCTeM), MIB, 131 Princess Street, Manchester, M1 7DN, UK; 3 Department of Computer Science, The University of Tokyo, 7-3-1 Hongo, Bunkyo-ku, Tokyo, Japan

## Abstract

**Background:**

Previous studies of named entity recognition have shown that a reasonable level of recognition accuracy can be achieved by using machine learning models such as conditional random fields or support vector machines. However, the lack of training data (i.e. annotated corpora) makes it difficult for machine learning-based named entity recognizers to be used in building practical information extraction systems.

**Results:**

This paper presents an active learning-like framework for reducing the human effort required to create named entity annotations in a corpus. In this framework, the annotation work is performed as an iterative and interactive process between the human annotator and a probabilistic named entity tagger. Unlike active learning, our framework aims to annotate all occurrences of the target named entities in the given corpus, so that the resulting annotations are free from the sampling bias which is inevitable in active learning approaches.

**Conclusion:**

We evaluate our framework by simulating the annotation process using two named entity corpora and show that our approach can reduce the number of sentences which need to be examined by the human annotator. The cost reduction achieved by the framework could be drastic when the target named entities are sparse.

## Background

Named entities play a central role in conveying important domain specific information in text, and good named entity recognizers are often required in building practical information extraction systems. Previous studies have shown that automatic named entity recognition can be performed with a reasonable level of accuracy by using various machine learning models such as support vector machines (SVMs) or conditional random fields (CRFs) [1-3].

However, the lack of annotated corpora, which are indispensable for training machine learning models, makes it difficult to broaden the scope of text mining applications. In the biomedical domain, for example, several annotated corpora such as GENIA [4], PennBioIE [5], and GENETAG [6] have been created and made publicly available, but the named entity categories annotated in these corpora are tailored to their specific needs and not always sufficient or suitable for text mining tasks that other researchers need to carry out.

*Active learning *is a framework which can be used for reducing the amount of human effort required to create a training corpus [7-10]. In active learning, samples that need to be annotated by the human annotator are picked up from a big pool of samples by a machine learning model in an iterative and interactive manner, considering the informativeness of the samples. It has been shown that, compared to random sampling, active learning can often drastically reduce the amount of training data necessary to achieve the same level of performance. The effectiveness of active learning has been demonstrated in several natural language processing tasks including named entity recognition.

The problem with active learning, however, is that the resulting annotated data is dependent on the machine learning algorithm and the sampling strategy employed, because active learning annotates only a *subset *of the samples in the given corpus. This sampling bias is not a serious problem if one is to use the annotated corpus only for their own machine learning purpose and with the same machine learning algorithm. However, the existence of bias is not desirable if one wants the corpus to be used by other applications or researchers. For the same reason, active learning approaches cannot be used to enrich an existing linguistic corpus with a new named entity category.

In this paper, we present a framework that enables one to make named entity annotations for a given corpus with a reduced cost. Unlike active learning approaches, our framework aims to annotate *all *named entities of the target category contained in the corpus. Obviously, if we were to ensure 100% coverage of annotation, there is no way of reducing the annotation cost, i.e. the human annotator has to go through every sentence in the corpus. However, we show in this paper that it is possible to reduce the cost by slightly relaxing the requirement for the coverage, and the reduction can be drastic when the target named entities are sparse.

We should note here that the purpose of this paper is not to claim that our approach is superior to existing active learning approaches. The goals are different – while active learning aims at optimizing the performance of the resulting machine learning-based tagger, our framework aims to help develop an unbiased named entity-annotated corpus.

## Methods

### Annotating named entities by dynamic sentence selection

**Figure 1:**
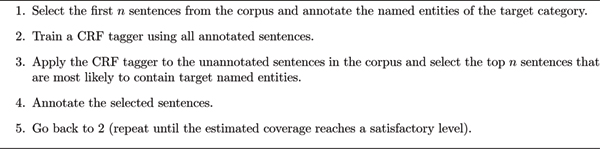
Annotating named entities by dynamic sentence selection.

Figure 1 shows the overall flow of our annotation framework. The framework is an iterative process between the human annotator and a named entity tagger based on CRFs. In each iteration, the CRF tagger is trained using all annotated sentences available and is applied to the unannotated sentences to select sentences that are likely to contain named entities of the target category. The selected sentences are then annotated by the human annotator and moved to the pool of annotated sentences.

This overall flow of annotation framework is very similar to that of active learning. In fact, the only differences are the criterion of sentence selection and the fact that our framework uses the estimated coverage as the stopping condition. In active learning, sentences are selected according to their informativeness to the machine learning algorithm. Our approach, in contrast, selects sentences that are most likely to contain named entities of the target category. The next section elaborates on how to select such sentences using the output of the CRF-based tagger.

The other key in this annotation framework is when to stop the annotation work. If we repeat the process until all sentences are annotated, then obviously there is no merit of using this approach. We show in the next section that we can quite accurately estimate how many of the entities in the corpus are already annotated and use this estimated coverage as the stopping condition.

### Selecting sentences using the CRF tagger

Our annotation framework takes advantage of the ability of CRFs to output multiple probabilistic hypotheses. This section describes how we obtain named entity candidates and their probabilities from CRFs in order to compute the expected number of named entities contained in a sentence.

We should note that one could use other machine learning algorithms for this task as long as they can produce probabilistic output. For example, maximum entropy Markov models are a possible alternative. We have chosen the CRF model because it is currently one of the best models for named entity recognition and there are efficient algorithms to compute marginal probabilities and N-best sequences in CRFs.

#### The CRF tagger

CRFs [11] can be used for named entity recognition by representing the spans of named entities using the "BIO" tagging scheme, in which 'B' represents the beginning of a named entity, 'I' the inside, and 'O' the outside (See Table 1 for example). This representation converts the task of named entity recognition into a sequence tagging task.

**Table 1:** N-best sequences output by the CRF tagger

Probability	Transcription	factor	GATA-1	and	the	estrogen	receptor
0.677	B	I	O	O	O	O	O
0.242	B	I	O	O	O	B	I
0.035	O	O	O	O	O	O	O
0.012	B	I	I	O	O	O	O
0.009	B	I	I	O	O	B	I
:	:	:	:	:	:	:	:

A linear chain CRF defines a single log-linear probabilistic distribution over the possible tag sequences **y **for a sentence **x**:

$$p(y|x)=\frac{1}{Z(x)}\exp\sum_{t=1}^{T}\sum_{k=1}^{K}\lambda_{k}f_{k}(t,y_{t},y_{t-1},x_{t}),$$

where *f*_*k*_(*t*, *y*_*t*_, *y*_*t*-1_, **x**_**t**_) is typically a binary function indicating the presence of feature *k*, *λ*_*k *_is the weight of the feature, and *Z*(*X*) is a normalization function:

$$Z(x)=\sum_{y}\exp\sum_{t=1}^{T}\sum_{k=1}^{K}\lambda_{k}f_{k}(t,y_{t},y_{t-1},x_{t}).$$

This modeling allows us to define features on states ("BIO" tags) and edges (pairs of adjacent "BIO" tags) combined with observations (e.g. words and part-of-speech (POS) tags).

The weights of the features are determined in such a way that they maximize the conditional log-likelihood of the training data: $ℒ(\theta )=\sum_{i=1}^{N}\log p_{\theta }(y^{\left(i\right)}|x^{\left(i\right)})$. In the actual implementation, we also used the L2-norm penalty term to avoid overfitting of the model to the training data. We used the L-BFGS algorithm [12] to compute the parameters.

Table 2 shows the feature templates used in the CRF tagger. We used unigrams of words/POS tags, and prefixes and suffixes of the current word. The current word is also normalized by lowering capital letters and converting all numerals into '#', and used as a feature. We created a word shape feature from the current word by converting consecutive capital letters into 'A', small letters 'a', and numerals '#'.

**Table 2:** Feature templates used in the CRF tagger

Word Unigram	*w*_*i*_, *w*_*i*-1_, *w*_*i*+1_	&*y*_*i*_
POS Unigram	*p*_*i*_, *p*_*i*-1_, *p*_*i*+1_	&*y*_*i*_
Prefix, Suffix	prefixes of *w*_*i*_	&*y*_*i*_
	suffixes of *w*_*i*_	&*y*_*i*_
	(up to length 3)	
Normalized Word	N(*w*_*i*_)	&*y*_*i*_
Word Shape	S(*w*_*i*_)	&*y*_*i*_
Tag Bi-gram	true	&*y*_*i*-1_*y*_*i*_

#### Computing the expected number of named entities

To select sentences that are most likely to contain named entities of the target category, we need to obtain the *expected number *of named entities contained in each sentence. CRFs are well-suited for this task as the output is fully probabilistic – one can easily obtain probabilistic information on possible tag sequences using established algorithms (i.e. A* and forwrad-backward algorithms).

Suppose, for example, that the sentence is "Transcription factor GATA-1 and the estrogen receptor". Table 1 shows an example of the 5-best sequences output by the CRF tagger. The sequences are represented by the aforementioned "BIO" representation. For example, the first sequence indicates that there is one named entity 'Transcription factor' in the sequence. By summing up these probabilistic sequences, we can compute the probabilities for possible named entities in a sentence. From the five sequences in Table 1, we obtain the following three named entities and their corresponding probabilities.

'Transcription factor' (0.677 + 0.242 = 0.916)

'estrogen receptor' (0.242 + 0.009 = 0.251)

'Transcription factor GATA-1' (0.012 + 0.009 = 0.021)

The expected number of named entities in this sentence can then be calculated as 0.916 + 0.251 + 0.021 = 1.188.

In this example, we used 5-best sequences as an approximation to all possible sequences needed to compute the exact expected number of entities. One possible way to achieve a good approximation is to use a large *N *for *N*-best sequences, but there is a simpler and more efficient way, which directly produces the exact expected number of entities. Recall that named entities are represented with the "BIO" tags. Since one entity always contains one "B" tag, we can compute the number of expected entities by simply summing up the marginal probabilities for the "B" tags on all tokens in the sentence. The marginal probabilities on each token can be computed by the forward-backward algorithm. This is normally more efficient than computing *N*-best sequences for a large *N*. For efficient implementation of the forward-backward algorithm, see [13].

Once we compute the expected number of entities for every unannotated sentence in the corpus, we sort the sentences in descending order of the expected number of entities and choose the top *n *sentences to be presented to the human annotator.

### Coverage estimation

To ensure the quality of the resulting annotated corpus, it is crucial to be able to know the current coverage of annotation at each iteration in the annotation process. To compute the coverage, however, one needs to know the total number of target named entities in the corpus. The problem is that it is not known until all sentences are annotated.

In this paper, we solve this dilemma by using an estimated value for the total number of entities. Then, the estimated coverage can be computed as follows:

(1) $$\left(estimated\text{\_}coverage\right)=\frac{m}{m+\sum_{i\in U}E_{i}}$$

where *m *is the number of entities actually annotated so far and *E*_*i *_is the expected number of entities in sentence *i*, and *U *is the set of unannotated sentences in the corpus. At any iteration, *m *is always known and *E*_*i *_is obtained from the output of the CRF tagger as explained in the previous section.

## Results and discussion

We carried out experiments to see how our method can improve the efficiency of annotation process for sparse named entities. We evaluate our method by simulating the annotation process using existing named entity corpora. In other words, we use the gold-standard annotations in the corpus as the annotations that would be made by the human annotator during the annotation process.

### Corpus

We used two named entity corpora for the experiments. One is the training data provided for the CoNLL-2003 shared task [1], which consists of 14,041 sentences and includes four named entity categories (LOC, MISC, ORG, and PER) for the general domain. The other is the training data provided for the NLPBA shared task [14], which consists of 18,546 sentences and five named entity categories (DNA, RNA, cell_line, cell_type, and protein) for the biomedical domain. This corpus is created from the GENIA corpus [4] by merging the original fine-grained named entity categories.

Table 3 shows statistics of the named entities included in the corpora. The first column shows the number of named entities for each category. The second column shows the number of the sentences that contain the named entities of each category. We can see that some of the named entity categories are very sparse. For example, named entities of "RNA" appear only in 4.4% of the sentences in the corpus. In contrast, named entities of "protein" appear in more than 70% of the sentences in the corpus.

**Table 3:** Statistics of named entities

	# Entities	Sentences (%)
CoNLL: LOC	7,140	5,127 (36.5%)
CoNLL: MISC	3,438	2,698 (19.2%)
CoNLL: ORG	6,321	4,587 (32.7%)
CoNLL: PER	6,600	4,373 (31.1%)

GENIA: DNA	2,017	5,251 (28.3%)
GENIA: RNA	225	810 (4.4%)
GENIA: cell_line	835	2,880 (15.5%)
GENIA: cell_type	1,104	5,212 (28.1%)
GENIA: protein	5,272	13,040 (70.3%)

In the experiments reported in the following sections, we do not use the "protein" category because there is little merit of using our framework when most sentences are relevant to the target category.

### Accelerated annotation

We carried out eight sets of experiments, each of which corresponds to one of those named entity categories shown in Table 3 (excluding the "protein" category). The number of sentences selected in each iteration (the value of *n *in Figure 1) was set to 100 throughout all experiments.

**Figure 2:**
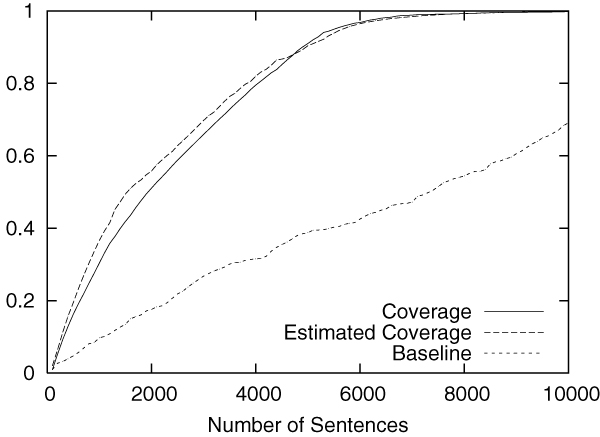
Annotation of LOC in the CoNLL corpus.

**Figure 3:**
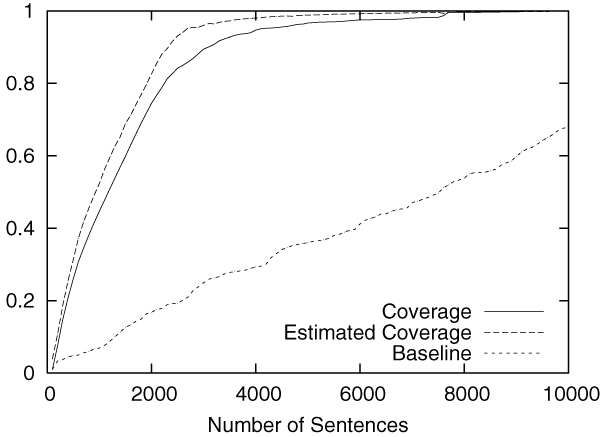
Annotation of MISC in the CoNLL corpus.

**Figure 4:**
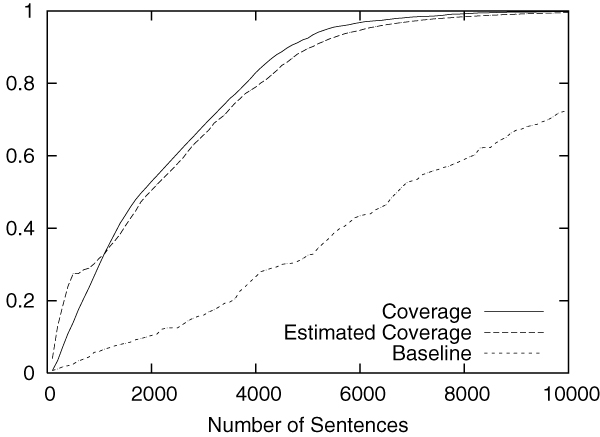
Annotation of ORG in the CoNLL corpus.

**Figure 5:**
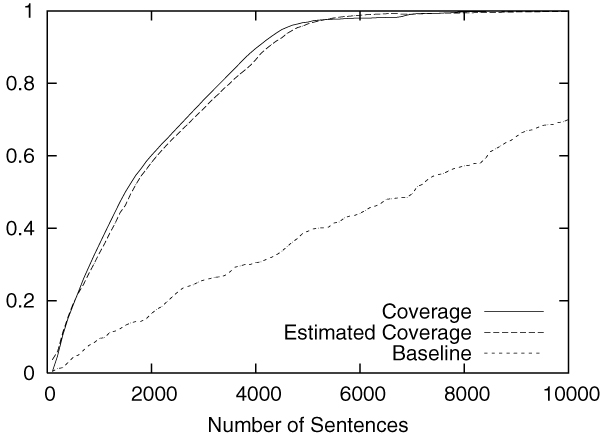
Annotation of PER in the CoNLL corpus.

Figures 2 to 5 show the results obtained on the CoNLL corpus. The figures show how the coverage increases as the annotation process proceeds. The x-axis shows the number of annotated sentences.

Each figure contains three lines. The normal line represents the coverage actually achieved, which is computed as follows:

(2) $$\left(coverage\right)=\frac{entities\text{\_}annotated}{total\text{\_}number\text{\_}of\text{\_}entities}.$$

The dashed line represents the coverage estimated by using Equation 1. For the purpose of comparison, the dotted line shows the coverage achieved by the baseline annotation strategy in which sentences are selected sequentially from the beginning to the end in the corpus.

The figures clearly show that our method can drastically accelerate the annotation process in comparison to the baseline annotation strategy. The improvement is most evident in Figure 3, in which named entities of the category "MISC" are annotated.

We should also note that coverage estimation was surprisingly accurate. In all experiments, the difference between the estimated coverage and the real coverage was very small. This means that we can safely use the estimated coverage as the stopping condition for the annotation work.

**Figure 6:**
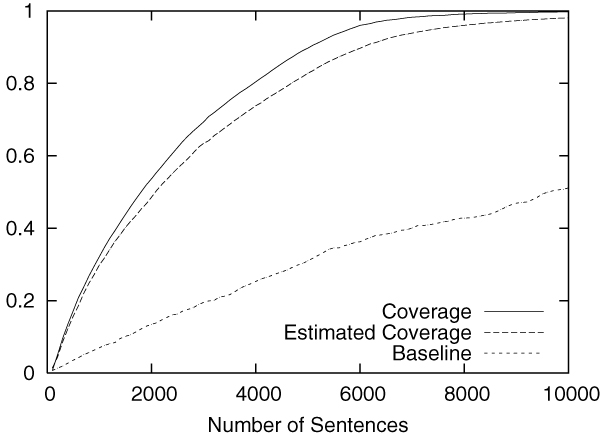
Annotation of DNA in the GENIA corpus.

**Figure 7:**
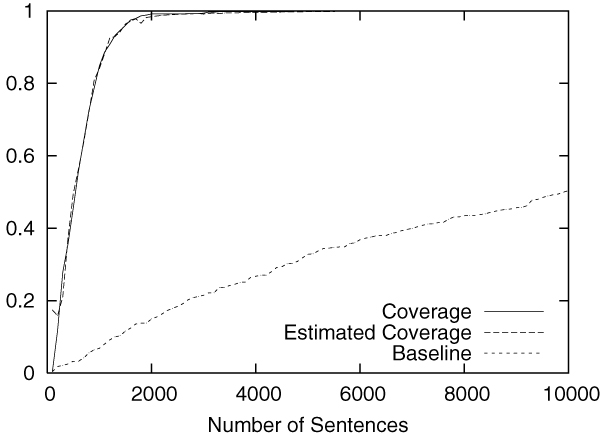
Annotation of RNA in the GENIA corpus.

**Figure 8:**
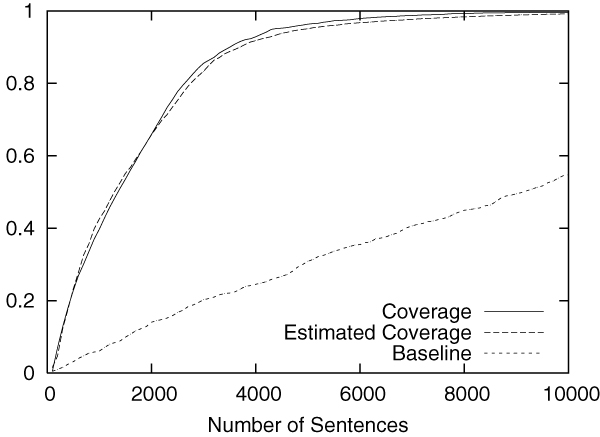
Annotation of cell_line in the GENIA corpus.

**Figure 9:**
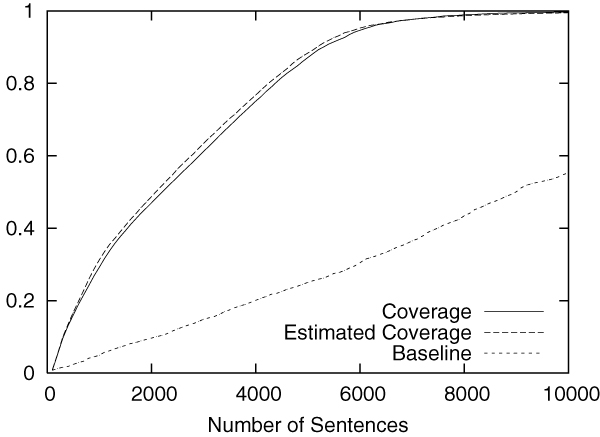
Annotation of cell_type in the GENIA corpus.

Figures 6 to 9 show the experimental results on the GENIA data. The figures show the same characteristics observed in the CoNLL data. The acceleration by our framework was most evident for the "RNA" category. Table 4 shows how much we can save the annotation cost if we stop the annotation process when the estimated coverage reaches 99%. The first column shows the coverage actually achieved, and the second and third columns show the number and percentage of the sentences annotated in the corpus. This result shows that, on average, we can achieve a coverage of 99.0% by annotating 52.4% of the sentences in the corpus. In other words, we could roughly halve the annotation cost by accepting the missing rate of 1.0%.

**Table 4:** Coverage achieved when the estimated coverage reached 99%

	Coverage	# Sentences Annotated	Percentage in the Corpus
CoNLL: LOC	99.1%	7,600	54.1%
CoNLL: MISC	96.9%	5,400	38.5%
CoNLL: ORG	99.7%	8,900	63.4%
CoNLL: PER	98.0%	6,200	44.2%

GENIA: DNA	99.8%	11,900	64.2%
GENIA: RNA	99.2%	2,500	13.5%
GENIA: cell_line	99.6%	9,400	50.7%
GENIA: cell_type	99.3%	8,600	46.4%

Average	99.0%	-	52.4%

As expected, the cost reduction was most drastic when "RNA", which is the most sparse named entity category (see Table 3), was targeted. The cost reduction was more than seven-fold. These experimental results confirm that our annotation framework is particularly useful when applied to sparse named entities.

One of the potential problems with this kind of active learning-like framework is the computation time required to retrain the tagger at each iteration. Since the human annotator has to wait while the tagger is being retrained, the computation time required for retraining the tagger should not be very long. Table 5 shows the time elapsed in the experiments. We used AMD Opteron 2.2 GHz servers for the experiments and our CRF tagger is implemented in C++. In our experiments, the worst case (i.e. DNA) required 443 seconds for retraining the tagger at the last iteration, but in most cases the training time for each iteration was kept under several minutes. We used the BFGS algorithm for training the CRF model in this work, but it is probably possible to further reduce the training time by using more recent parameter estimation algorithms such as exponentiated gradient algorithms [15].

**Table 5:** Time elapsed when the estimated coverage reached 99%

	Cumulative Time (second)	Last Interval (second)
CoNLL: LOC	3,362	92
CoNLL: MISC	1,818	61
CoNLL: ORG	5,201	104
CoNLL: PER	2,300	75

GENIA: DNA	33,464	443
GENIA: RNA	822	56
GENIA: cell_line	15,870	284
GENIA: cell_type	13,487	295

### Suggesting annotation candidates

In the previous section, we discussed how much we can reduce the number of sentences that need to be examined by the annotator, but we did not discuss the annotation cost for individual sentences presented to the annotator. Here we present some experimental results to show that the annotation cost for each sentence could be reduced if we take advantage of the N-best sequences output from the CRF tagger by using the idea presented in [16].

The idea is to present the N-best sequences from the CRF tagger to the annotator as the annotation candidates, so that the annotator does not have to make the annotation for the sentence from scratch. In other words, all (s)he has to do is selecting the correct annotation for the sentence from the list of likely annotations suggested by the system.

The effectiveness of this approach is almost exclusively dependent on the quality of the annotation candidates generated by the CRF tagger. To investigate their quality, we carried out additional experiments using the GENIA corpus and the RNA category.

Table 6 shows the result. The first three columns show the number of iteration, the actual coverage achieved, and the estimated coverage respectively. These three columns simply show the same data presented in Figure 7. The fourth column shows the percentage of the sentences that contained at least one target named entity, among the 100 sentences presented in each iteration.

**Table 6:** Detailed results of the annotation process for GENIA:RNA

Iteration	Coverage	Estimated Coverage	Relevant Sentences	Coverage of Suggested Annotation	Average Rank of Suggested Annotation
1	0.4%	17.4%	85%	86%	2.64
2	11.8%	15.9%	90%	82%	2.12
3	27.9%	21.1%	58%	83%	2.54
4	35.6%	37.3%	87%	94%	1.48
5	45.4%	49.4%	89%	96%	1.50
6	55.6%	56.3%	79%	96%	1.65
7	64.2%	63.7%	74%	98%	1.60
8	72.6%	72.1%	55%	95%	2.00
9	78.7%	80.9%	56%	98%	1.78
10	84.8%	84.1%	36%	99%	1.54
11	88.6%	88.1%	18%	99%	1.48
12	90.7%	92.5%	21%	98%	1.31
13	93.2%	92.9%	12%	98%	1.21
14	94.6%	94.3%	12%	100%	1.24
15	96.0%	96.4%	12%	99%	1.27
16	97.5%	97.2%	4%	99%	1.03
17	97.9%	97.8%	5%	99%	1.11
18	98.6%	96.6%	2%	100%	1.15
19	98.8%	98.2%	3%	99%	1.02
20	99.2%	98.4%	0%	100%	1.00
21	99.2%	98.6%	0%	100%	1.00
22	99.2%	98.8%	0%	100%	1.00
23	99.2%	98.9%	0%	100%	1.00
24	99.2%	99.0%	0%	100%	1.00
25	99.2%	99.1%	0%	100%	1.00

In this experiment, we assumed that the CRF tagger generates 10 annotation candidates for each sentence. The fifth column in Table 6 shows the percentage of the cases where the correct annotation for the sentence was actually included in the candidates. The sixth column shows the average rank of the correct annotation in the candidates. The experimental results were promising – the correct annotation was included in the top 10 candidates in most cases and they are usually highly ranked in the list.

### Enriching an existing corpus

In the experiments presented in the previous sections, we assumed that the annotation work for each named entity category is carried out independently from other categories. However, there are cases where we can take advantage of the information about named entities of other categories.

Let us suppose a situation where we want to enrich an existing named entity corpus with a new named entity category. If named entities are not allowed to overlap, we can rule out the text regions that are already covered by the existing named entity categories when computing the expected numbers of target named entities, which should improve the accuracy of estimated coverage and lead to improved efficiency of annotation work.

We carried out another set of experiments to simulate this kind of situation. We used the same eight categories that were used in the previous experiments, but we assumed the existence of the named entities of the other categories. For example, when we ran the experiments for the LOC category in the CoNLL corpus, we assumed that the corpus was already annotated with the other three categories (i.e. MISC, ORG, and PER) and named entities were not allowed to overlap.

The results are shown in Table 7. As expected, the numbers of sentences that needed to be examined were much smaller than those in the previous experiments shown in Table 4.

**Table 7:** Coverage achieved when the estimated coverage reached 99% (assuming the named entities of the other categories are already annotated in the corpus)

	Coverage	# Sentences Annotated	Percentage in the Corpus
CoNLL: LOC	98.5%	5,500	39.2%
CoNLL: MISC	95.0%	3,200	22.8%
CoNLL: ORG	99.0%	5,400	38.5%
CoNLL: PER	97.9%	4,700	33.5%

GENIA: DNA	99.6%	8,200	44.2%
GENIA: RNA	99.5%	1,800	9.7%
GENIA: cell_line	99.3%	5,000	27.0%
GENIA: cell_type	99.2%	7,000	37.7%

Average	98.5%	-	31.6%

## Discussion and related work

Our annotation framework is, by definition, not something that can ensure a coverage of 100%. The seriousness of a missing rate of, for example, 1% is not entirely clear – it depends on the application and the purpose of annotation. In general, however, it is hard to achieve a coverage of 100% in real annotation work even if the human annotator scans through all sentences, because there is often ambiguity in deciding whether a particular named entity should be annotated or not. Previous studies report that inter-annotator agreement rates with regards to gene/protein name annotation are f-scores around 90% [17,18]. We believe that the missing rate of 1% can be an acceptable level of sacrifice, given the cost reduction achieved and the unavoidable discrepancy made by the human annotator.

At the same time, we should also note that our framework could be used in conjunction with existing methods for semi-supervised learning to improve the performance of the CRF tagger, which in turn will improve the coverage. It is also possible to improve the performance of the tagger by using external dictionaries or using more sophisticated probabilistic models such as semi-Markov CRFs [19]. These enhancements should further improve the coverage, keeping the same degree of cost reduction.

The idea of improving the efficiency of annotation work by using automatic taggers is certainly not new. Tanabe et al. [6] applied a gene/protein name tagger to the target sentences and modified the results manually. Culotta and McCallum [16] proposed to have the human annotator select the correct annotation from multiple choices produced by a CRF tagger for each sentence. Tomanek et al. [20] discuss the reusability of named entity-annotated corpora created by an active learning approach and show that it is possible to build a corpus that is useful to different machine learning algorithms to a certain degree.

The limitation of our framework is that it is useful only when the target named entities are sparse because the upper bound of cost saving is limited by the proportion of the relevant sentences in the corpus. Our framework may therefore not be suitable for a situation where one wants to make annotations for named entities of many categories simultaneously (e.g. creating a corpus like GENIA from scratch). In contrast, our framework should be useful in a situation where one needs to modify or enrich named entity annotations in an existing corpus, because the target named entities are almost always sparse in such cases. We should also note that named entities in full papers, which recently started to attract much attention, tend to be more sparse than those in abstracts.

## Conclusion

We have presented a simple but powerful framework for reducing the human effort for making name entity annotations in a corpus. The proposed framework allows us to annotate *almost *all named entities of the target category in the given corpus without having to scan through all the sentences. The framework also allows us to know when to stop the annotation process by consulting the estimated coverage of annotation.

Experimental results demonstrated that the framework can reduce the number of sentences to be annotated almost by half, achieving a coverage of 99.0%. Our framework was particularly effective when the target named entities were very sparse.

Unlike active learning, this work enables us to create a named entity corpus that is free from the sampling bias introduced by the active learning strategy. This work will therefore be especially useful when one needs to enrich an existing linguistic corpus (e.g. WSJ, GENIA, or PennBioIE) with named entity annotations for a new semantic category.

## Competing interests

The authors declare that they have no competing interests.

## Authors' contributions

YT developed the algorithm, carried out the experiments and drafted the manuscript. JT and SA conceived the study and participated in its design and coordination. All authors read and approved the final manuscript.
